# Toxicological impact of benzo[a]pyrene on esophageal cancer: an integrated analysis via network toxicology, machine learning, and molecular docking

**DOI:** 10.3389/fimmu.2026.1881352

**Published:** 2026-07-15

**Authors:** Xuyan Lan, Zuqiang Huang, Yukun Lin, Xiaoyu Sun, Binghan Guo, Genglin Li, Jintao Wang, Jinlan Lin, Lihuan Zhu, Tianxing Guo

**Affiliations:** 1 Shengli Clinical Medical College, Fujian Medical University, Fuzhou, China; 2 Department of Thoracic Oncology, Fujian Cancer Hospital, Fuzhou, China; 3 Department of Thoracic Surgery, Fuzhou University Affiliated Provincial Hospital, Fuzhou, China

**Keywords:** benzo[a]pyrene, esophageal cancer, molecular docking, network toxicology, single-cell RNA sequencing

## Abstract

**Background:**

To investigate the mechanisms underlying benzo[a]pyrene-induced esophageal cancer (EC), and to screen and identify the key targets and biomarkers associated with benzo[a]pyrene-related EC.

**Methods:**

Potential targets of benzo[a]pyrene (BaP) were predicted using PharmMapper, SwissTargetPrediction, and ChEMBL databases, and were intersected with differentially expressed genes (DEGs) from the GEO database to screen candidate key genes. Subsequently, diagnostic models were constructed using 14 machine learning algorithms based on the identified key genes. Meanwhile, a prognostic model of key genes was constructed based on the TCGA esophageal cancer cohort, and the correlation between these key genes and tumor immune infiltration was further explored. Additional explainability was provided via SHAP analysis by determining the contributions of key features. Molecular docking was performed to verify the binding between BaP and core targets.

**Results:**

A total of 82 genes were identified as potential targets of EC induced by BaP. These key genes were found to be mainly involved in core tumor-related pathways, cell cycle regulation, MAPK signaling, and immune-inflammatory pathways, covering the crucial biological processes underlying malignant transformation of EC. Subsequently, 12 core genes (*ACOT9、ACOX3、AURKA、HMGCR、INHBA、MMP3、MSR1、SHC1、SORT1、MAOB、MMP13、CDK4*) were identified as key regulators by machine learning analysis. Among them, *SORT1* and *ACOX3* were significantly down-regulated, while *AURKA* and *MMP13* were markedly up-regulated (*P* < 0.05). The 12-gene prognostic model enables efficient prognostic stratification of esophageal cancer patients, and core genes are implicated in the remodeling of the esophageal cancer immunosuppressive microenvironment through the regulation of immune cell infiltration. Molecular docking revealed strong binding ability between BaP and target proteins.

**Conclusions:**

Bioinformatics analysis and molecular docking results revealed significant associations between BaP and 12 core esophageal cancer-related genes. BaP could stably bind to core proteins including *AURKA*, *CDK4*, *MMP13* and *INHBA*, which is potentially correlated with altered cell cycle, metabolic disorders and dysregulated tumor immune microenvironment in esophageal cancer. 12 core genes were identified via machine learning, which offers new perspectives for the interdisciplinary field of environmental toxicology and precision oncology and provides a foundation for the development of individualized therapeutic strategies.

## 1 Introduction

Esophageal cancer (EC) is a highly prevalent malignant gastrointestinal tumor worldwide. Its incidence and mortality rank among the highest of all malignant tumors, imposing a heavy burden on public health and patients (1, 2). Esophageal squamous cell carcinoma and adenocarcinoma are the main pathological types of EC. At present, the treatment of EC is still limited by molecular heterogeneity, chemotherapy resistance and other issues, and the prognosis remains unsatisfactory (3, 4). Therefore, elucidation of environmental risk factors and underlying carcinogenic molecular mechanisms of EC is of great theoretical and clinical significance for early screening, risk prevention and control, as well as precise diagnosis and treatment of this disease. Polycyclic aromatic hydrocarbons (PAHs) exposure is recognized as one of the important risk factors for the development of EC (5).

Benzo[a]pyrene (BaP) is the most representative pollutant among PAHs. It is classified as a Group 1 human carcinogen by the International Agency for Research on Cancer, and its carcinogenicity has been widely confirmed (6, 7). BaP originates from a wide range of sources and is mainly produced during the incomplete combustion of organic materials, including tobacco smoke, cooking fumes, industrial emissions, vehicle exhaust, as well as long-term consumption of barbecued, smoked, and fried foods (8–10). BaP is characterized by stable physicochemical properties and resistance to degradation. It can enter the human body through inhalation, ingestion of food and water, and dermal exposure, and exerts persistent toxic effects after long-term accumulation (11, 12).

Previous studies have confirmed that BaP exposure is significantly associated with the risk of multiple cancers, including lung, gastric, colorectal, ovarian, cervical and breast cancer. Its carcinogenic mechanisms mainly involve DNA damage and epigenetic alterations, aberrant activation of signaling pathways, inflammatory responses and oxidative stress (13–18). The most classic mechanism of BaP-induced carcinogenesis lies in the fact that reactive metabolites are generated after BaP is metabolically activated. These metabolites can interact with DNA, DNA adducts are formed, and mutations are induced in tumor suppressor genes or proto-oncogenes (19–21). However, other mechanisms may exist (22, 23). Furthermore, recent studies have shown that BaP may be involved in the tumorigenesis process by affecting the composition of the intestinal flora and mediating the microbe-host interaction (24). However, obvious deficiencies exist in the current research on BaP-induced EC, and in-depth systematic research is relatively limited.

The emergence of network toxicology has provided a comprehensive research framework for the analysis of toxicological effects, and integrated multi-omics analysis is utilized to systematically construct a multi-dimensional interaction network between chemical substances, biological targets and toxic pathways (25, 26). Taking the association between BaP exposure and EC as the core, network toxicology, multi-omics analysis, machine learning, and molecular simulation technologies are integrated in this study to systematically explore the molecular mechanisms of EC induced by BaP. In this study, core target genes of BaP-related EC will be screened by integrating public databases. A risk prediction model will be constructed and its clinical value will be evaluated. Combined with single-cell transcriptome data, the effects of BaP exposure on the remodeling of the EC immune microenvironment and cell heterogeneity will be analyzed. The purpose of this study is to fill the gap in the research on the systematic mechanism of EC induced by BaP, explore potential correlated core targets and signaling pathways involved in BaP-associated esophageal carcinogenesis, and provide a basis for the early risk prediction, stratified treatment and precise prevention and control of EC.

## 2 Materials and methods

### 2.1 Acquisition and collection of disease targets

Three esophageal cancer transcriptomic datasets (GSE157804, GSE161533, and GSE20347) were curated from the Gene Expression Omnibus (GEO) database of the National Center for Biotechnology Information (NCBI) for analysis. To reduce batch effects, a multi-stage standardization strategy was adopted for data preprocessing. First, potential confounding factors were adjusted using the surrogate variable analysis (SVA) method in the discovery cohort. Subsequently, residual batch effects were further adjusted using a parametric empirical Bayes ComBat algorithm implemented in the sva R package. After standardized processing, principal component analysis (PCA) was employed to reduce the dimensionality of data. To quantitatively assess the elimination of batch heterogeneity, we calculated the variance explained by the top two principal components (PC1 and PC2) before and after correction. Two statistical indicators were additionally applied to quantify integration efficiency: permutational multivariate ADONIS analysis with 999 permutations to measure the proportion of transcriptional variation explained by batch labels, and average silhouette coefficient to evaluate the degree of sample aggregation grouped by dataset origin.

### 2.2 Acquisition of the chemical composition and its action targets of BaP

Systematic analysis of BaP was performed by integrating multisource databases. The physicochemical properties and biological parameters were obtained through systematic retrieval from PubMed. The standard 2D structure descriptor (SMILES: C1=CC=C2C3= C4C(=CC2=C1)C=CC5=C4C(=CC=C5)C=C3) was derived from the PubChem database. A three-step prediction strategy was adopted for potential target identification, involving ChEMBL (https://www.ebi.ac.uk/chembl/), SwissTargetPrediction (http://swisstargetprediction.ch/) and PharmMapper (https://lilab-ecust.cn/pharmmapper/index.html). All predicted targets were restricted to the human proteome.

### 2.3 Differential gene expression analysis

Differential expression analysis of transcriptomic data was performed using the limma package in R software(version 4.5.0). Differentially expressed genes (DEGs) were identified with thresholds of false discovery rate (FDR)-adjusted *P* < 0.05 and |log2FC|>0.585 (1.5 fold change). The results of differential analysis were visualized via the ggplot2 package.

### 2.4 Identify disease targets associated with BaP

Intersection analysis between DEGs and BaP-predicted potential targets identified key candidate targets, and these overlapping genes were defined as core BaP-related disease targets, visualized by Venn diagrams.

### 2.5 Protein-protein interaction network construction

The filtered intersecting genes were imported into the STRING database (https://cn.string-db.org/) to construct the PPI network. A minimum required interaction score of 0.4 (medium confidence) was set as the threshold to ensure the reliability of the results. The PPI network was visualized and analyzed using Cytoscape 3.9.1 software, and network characteristics were evaluated with topological methods to screen and identify potential hub genes.

### 2.6 Functional enrichment analysis

To systematically elucidate the potential molecular mechanisms of BaP in the occurrence and development of esophageal cancer, Gene Ontology (GO) analysis was performed on the screened key targets using the clusterProfiler package in R software, covering biological processes (BP), cellular components (CC), and molecular functions (MF). The KEGG gene set (c2.cp.kegg_legacy) from the MSigDB database was used, and hypergeometric distribution test was employed to calculate the enrichment significance (P < 0.05). The ggplot2 package of R software was used for the visualization.

### 2.7 Machine learning algorithms

To systematically screen for potential diagnostic markers of BaP associated with EC, the batch-corrected dataset was used as the discovery cohort, while the other three independent datasets (GSE23400, GSE26886, and GSE38129) were employed as external validation cohorts. During the model training phase, multiple machine learning algorithms were utilized to evaluate their performance, including Elastic Net regression (λ=0.1), Lasso regression (λ=0.05), Ridge regression (λ=1.0), support vector machine (SVM, C = 1.0, γ=0.01), linear discriminant analysis (LDA), gradient boosting machine (GBM, learning rate=0.1, number of trees=100), random forest (RF, number of trees=200), and XGBoost (XGB, learning rate=0.01, number of trees=150). These models were trained on the training set, and hyperparameters were optimized through cross-validation. In the model evaluation phase, the test set was used to calculate the AUC value of each model (with a threshold set at 0.7) to measure its classification performance. Finally, the AUC value of each model was calculated using the RunEval function, and a heatmap was generated using the SimpleHeatmap function to visualize the performance of each model. The model with the highest AUC value was selected as the final optimal model to determine the candidate core genes.

### 2.8 Validation of the TCGA prognostic model

Gene expression profiles and clinical data of the esophageal squamous cell carcinoma (ESCC) cohort were downloaded from TCGA database. A total of 12 core genes were enrolled to construct the prognostic model. According to the expression levels of the 12 core genes, a multivariate Cox proportional hazards regression model was established, and the risk score was calculated for each patient. To achieve optimal prognostic stratification, the surv_cutpoint function in the R survminer package was used to determine the optimal cutoff value. Kaplan-Meier(KM) survival curves combined with the log-rank test were applied to compare survival differences between different risk groups. Time-dependent ROC curves were utilized to evaluate the predictive performance of the risk score model, and the area under the curve (AUC) was used to quantify the predictive accuracy. All statistical analyses were performed using R software.

### 2.9 Immune infiltration analysis in the TCGA cohort

Based on the TCGA transcriptomic data of EC, the expression profiles of 12 core genes and CIBERSORT-derived abundance data of immune cell infiltration were integrated. After sample matching, Spearman’s correlation analysis was performed to investigate the associations between the expression levels of the 12 core genes and tumor-infiltrating immune cells, and the internal correlations among different immune cell subtypes were also analyzed. The correlation characteristics of gene−immune interactions and interrelationships among immune cell subtypes were visualized using the linkET and pheatmap packages in R software. A P-value of less than 0.05 was defined as the threshold for statistically significant differences.

### 2.10 Model interpretation

In this study, the SHAP (SHapley Additive explanation) algorithm was introduced to conduct interpretability analysis of the model. SHAP values were assigned to each feature by this method to quantify their marginal contributions to the model’s predictive outcomes, thereby enabling quantitative assessment and visual interpretation of the influence degree of each feature in the prediction process.

### 2.11 Immunohistochemistry

Five patients with a history of long-term exposure to BaP (including long-term exposure to industrial emissions, tobacco smoke or fried foods) who underwent esophageal cancer surgery in the Department of Thoracic Surgery, Fuzhou University Affiliated Provincial Hospital, were collected retrospectively. Unified inclusion and exclusion criteria were established. Subjects with long-term exposure to cooking fumes, tobacco, grilled food or industrial dust were enrolled; those with other malignancies or benign esophageal lesions were excluded. Exposure types and approximate durations were recorded via questionnaires, while quantitative BaP metabolite detection was unavailable. IHC data from matched patients without relevant exposure were supplemented as controls. Paraffin sections of surgically resected esophageal cancer tissues and adjacent normal esophageal tissues from the enrolled patients were collected. Immunohistochemical detection was performed on the key variables (*AURKA* and *MMP13*) obtained from SHAP analysis. After antigen retrieval and endogenous peroxidase blocking, the sections were incubated with AURKA antibody (10297-1-AP, Proteintech) and MMP13 antibody (18165-1-AP, Proteintech), respectively, followed by secondary antibody incubation and DAB chromogenic staining. After scanning the stained sections, 5 high-power fields (×400) were randomly selected to evaluate the expression of AURKA and MMP13.

### 2.12 Single cell transcriptomic analysis

The single-cell transcriptome data used in this study were obtained from the corresponding GSE196756 dataset in the GEO database. The Seurat package in R language was employed for systematic quality control and preprocessing of the raw data, including the removal of low-quality cells, potential environmental RNA-contaminated cells, and doublets. On this basis, the data were normalized, followed by nonlinear dimensionality reduction and cell clustering analysis using UMAP. Fine division of cell subpopulations was achieved by constructing a clustering tree and selecting the optimal resolution parameter. Furthermore, the SingleR package was used in combination with the human reference transcriptome database to perform automatic annotation of cell types. Meanwhile, UMAP distribution plots, bubble plots, and violin plots were plotted for the key genes included in the diagnostic model to systematically display their expression patterns and distribution characteristics in different cell types.

### 2.13 Molecular docking analysis

To evaluate the potential interaction between BaP and the screened core targets, molecular docking analysis was performed in this study. The structure of BaP (SDF format) was obtained from the PubChem database, and the structural information of target proteins was retrieved from the corresponding entries in the UniProt database and further matched with protein structural data. Standardized preprocessing was conducted on protein structures, including the removal of crystalline water molecules and the addition of polar hydrogen atoms. The docking grid was centered on the predicted active sites, and its dimensions were reasonably adjusted according to ligand properties and potential binding modes. All molecular docking procedures were implemented using the CB-Dock2 online platform (http://183.56.231.194:8001/cb-dock2/index.php).

## 3 Results

### 3.1 Identification of BaP target proteins

**Figure 1:**
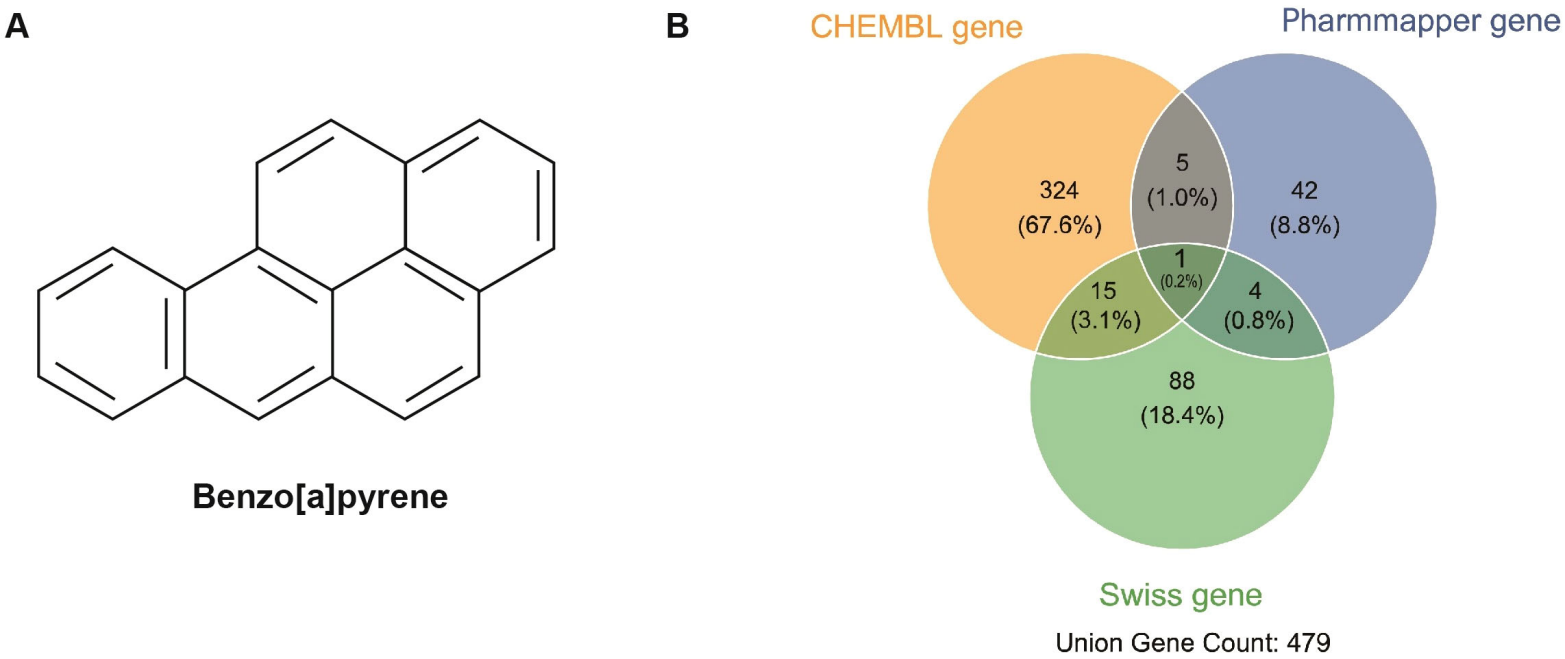
Identification of BaP target proteins. **(A)** Chemical structure of Bap. **(B)** Target prediction via CHEMBI, PharmMapper, and SwissTargetPrediction.

The molecular structure information of BaP was obtained from the PubChem database (**Figure 1A**). Subsequently, the potential targets of BaP were systematically predicted based on three complementary databases, namely ChEMBL, SwissTargetPrediction, and PharmMapper. After integrating data from different sources and removing duplicates, a total of 479 candidate targets were screened out (**Figure 1B**).

### 3.2 Identification of EC-associated target genes

**Figure 2:**
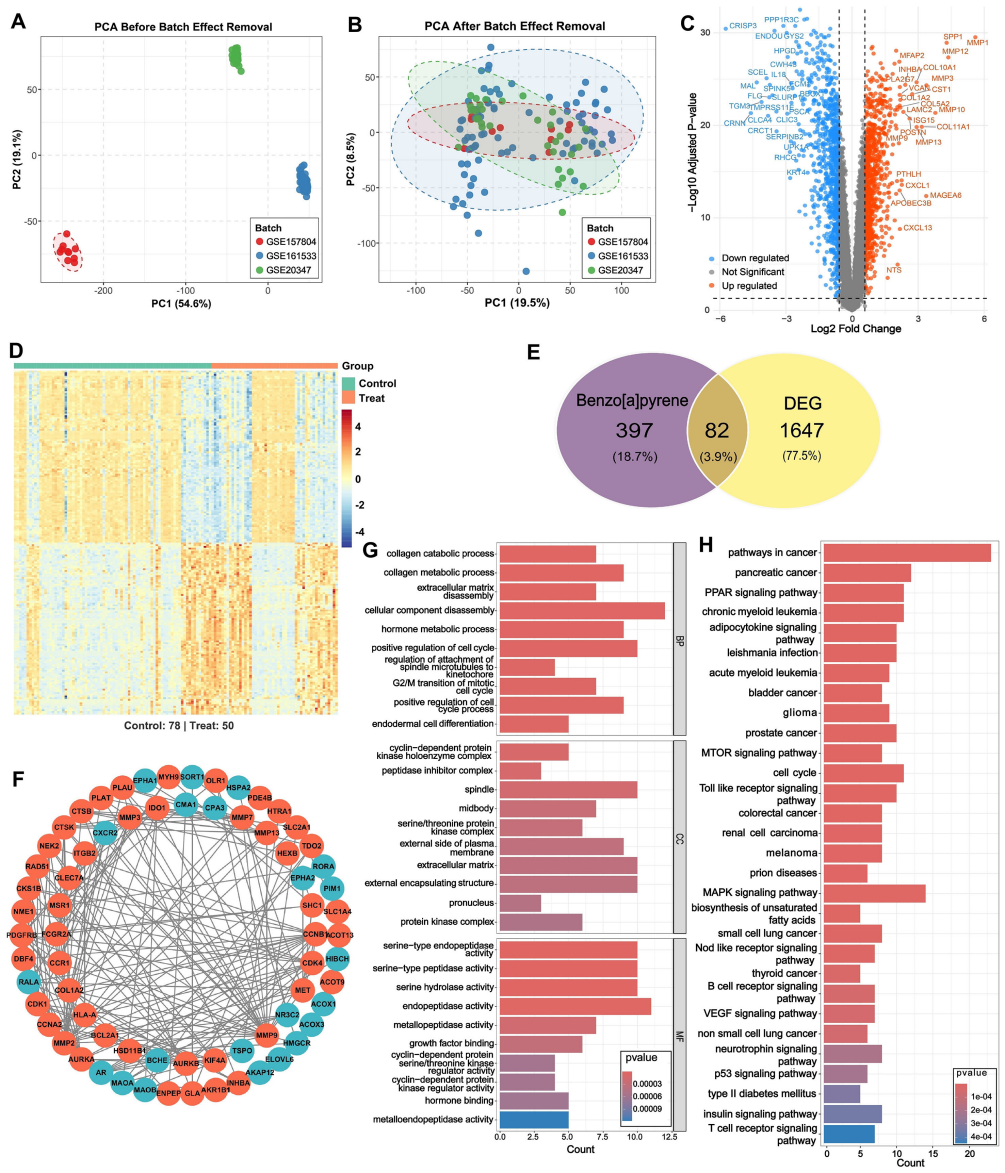
Screening and functional analysis of EC-associated target genes. **(A)** PCA showed distinct separation among the GSE157804, GSE161533, and GSE20347 datasets before batch correction. **(B)** After batch correction, the PCA results demonstrated that the distribution of each dataset tended to be consistent. **(C)** The volcano plot of DEGs. **(D)** Heatmap of DEGs displaying the expression patterns across different samples. **(E)** Venn diagram illustrated the intersection between BaP-related target genes and EC-related DEGs. **(F)** PPI network of the overlapping genes was constructed. **(G)** GO enrichment analysis of the overlapping genes. **(H)** KEGG enrichment analysis.

In this study, three datasets (GSE157804, GSE161533, and GSE20347) were integrated. PCA results showed that the sample distribution became more concentrated after normalization, and a clearer separation trend was observed between different groups (**Figures 2A, B**). Quantitative PCA decomposition revealed that PC1 and PC2 captured 56.0% and 17.5% of total transcriptional variance in uncorrected raw data; after ComBat batch adjustment, the variance contributions of PC1 and PC2 declined sharply to 23.8% and 11.7%, respectively, indicating substantial elimination of batch-associated sample segregation. ADONIS permutation analysis further quantified that batch origin accounted for 79.8% of total gene expression variation before correction, and this proportion was reduced to merely 3.0% following batch correction. In parallel, the average silhouette coefficient fell from 0.575 to -0.013, confirming nearly complete disappearance of batch-driven sample clustering and reliable integration of three independent esophageal cancer transcriptomic datasets. A total of 1729 genes with significant alterations in EC tissues were identified by differential expression analysis (**Figures 2C, D**). Furthermore, intersection analysis was performed between the above differentially expressed genes and BaP-predicted targets, and a total of 82 potential key targets were screened and obtained (**Figure 2E**). Protein-protein interaction (PPI) network analysis revealed that extensive and tight connections existed among these key target genes, forming a complex regulatory network centered on multiple hub genes (**Figure 2F**).

Functional enrichment analysis revealed that these key genes were significantly enriched in multiple biological processes and functional categories. Biological processes including cellular component disassembly and hormone metabolic process were identified. Cellular components such as spindle, midbody, and serine/threonine protein kinase complex were also enriched. Molecular functions including serine-type endopeptidase activity, endopeptidase activity, and serine hydrolase activity were observed (**Figure 2G**).

KEGG pathway enrichment analysis revealed that the key genes were most significantly enriched in cancer-related pathways, among which pathways in cancer contained the largest number of enriched genes, suggesting that these genes were widely involved in the core regulatory networks of tumorigenesis and development (**Figure 2H**). In addition, the MAPK, PPAR, cell cycle, and Toll-like receptor signaling pathways, as well as pro-proliferative pathways including p53, VEGF, and mTOR, and immune-related pathways were also highly enriched. These results demonstrated that the key targets of BaP-associated EC were mainly enriched in pathways related to tumorigenesis, cell cycle regulation, pro-proliferative signaling, immune inflammation, and metabolism, which were consistent with the results of GO analysis. These enrichment results indicated that these core genes were enriched in tumor-related biological processes and signaling pathways closely related to BaP toxic effects, revealing potential correlations between BaP exposure and esophageal malignant phenotypes.

### 3.3 Identification of core genes in BaP-induced EC

**Figure 3:**
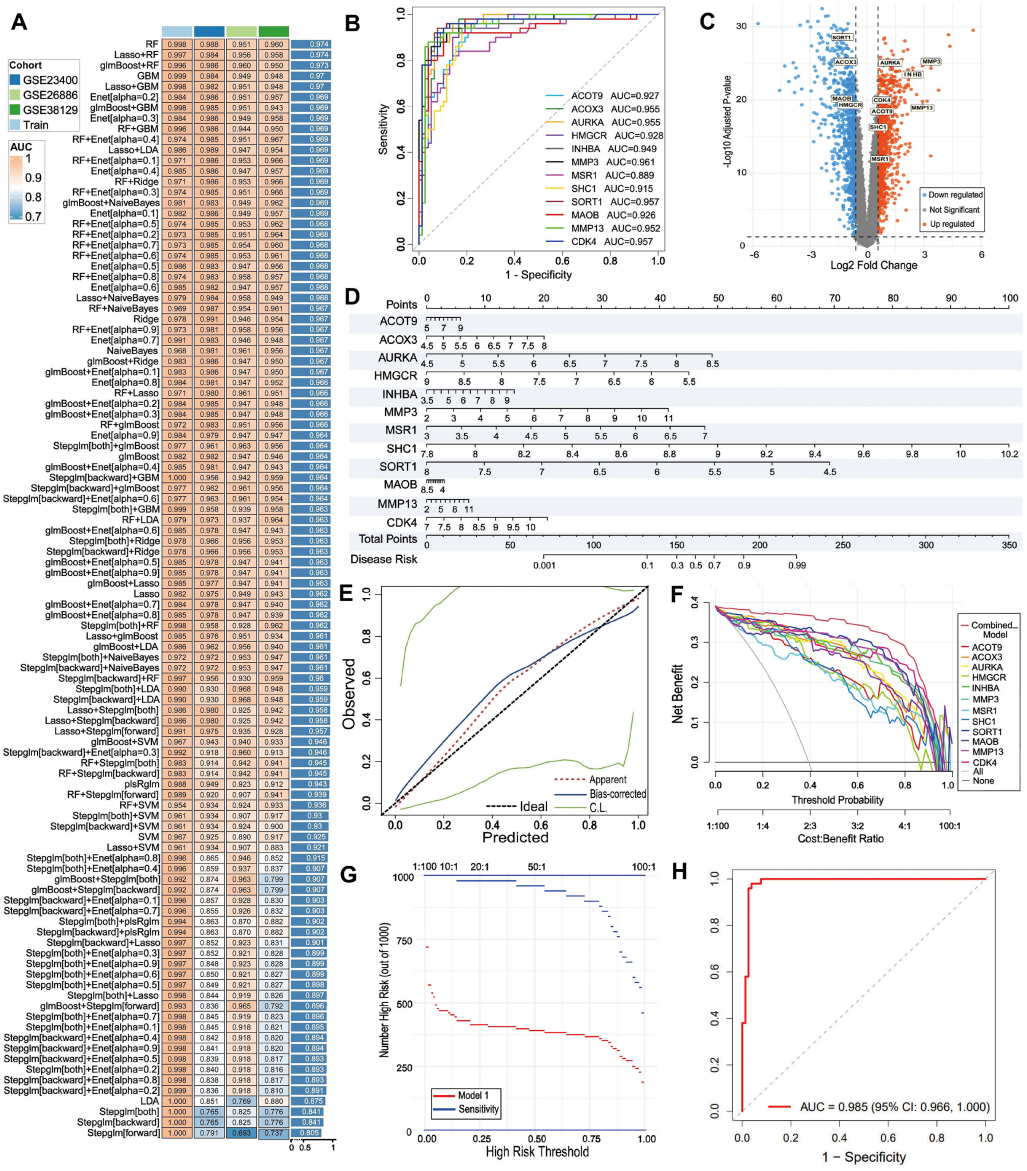
Screening of core genes related to BaP-associated EC and model construction. **(A)** Performance of different machine learning models in each dataset was compared, which was represented by the AUC. **(B)** ROC curves of the key genes (ACOT9, ACOX3, AURKA, HMGCR, INHBA, MMP3, MSR1, SHC1, SORT1, MAOB, MMP13, CDK4) were plotted. **(C)** Volcano plots of DEGs were generated, with core genes labeled. **(D)** A nomogram was constructed based on the core genes. total risk score was summed from each gene’s expression weight to calculate individual probability of BaP-related esophageal cancer. **(E)** Calibration curves of the nomogram were plotted to assess consistency between predicted risk and actual clinical outcomes. **(F)** DCA was performed to evaluate the clinical benefit of the model. **(G)** A decision curve analysis plot of cost-benefit versus threshold probability was generated to verify stable clinical applicability. **(H)** The ROC curve and AUC value of the comprehensive model score were presented, where higher AUC indicates stronger diagnostic discrimination ability.

Following systematic machine learning analysis of 82 candidate targets, 116 predictive models were constructed to screen key genes for BaP-associated EC. Among these, the combined model of Lasso and random forest (RF) exhibited the best performance, and the highest accuracy was achieved in both the training and validation datasets (**Figure 3A**). Based on this optimal model, 12 core genes were finally identified: *ACOT9*, *ACOX3*, *AURKA*, *HMGCR*, *INHBA*, *MMP3*, *MSR1*, *SHC1*, *SORT1*, *MAOB*, *MMP13*, *CDK4*. The diagnostic performance of these core genes was validated by ROC curve analysis, and all area under the curve (AUC) values were greater than 0.85 (**Figure 3B**). Meanwhile, the differential expression patterns of these genes in esophageal cancer were visualized by volcano plots (**Figure 3C**). On this basis, a nomogram model for risk prediction of BaP-associated esophageal cancer was established using the 12 core genes, by which individual cancer risk could be quantitatively evaluated according to the total score corresponding to the expression level of each gene (**Figure 3D**). The calibration curves indicated that good consistency was obtained between the predicted probabilities and the actual observations (**Figure 3E**). Furthermore, decision curve analysis (DCA) was performed to evaluate the clinical application value of the model. The results showed that the combined model conferred a higher net benefit than single gene indicators across a wide range of threshold probabilities (**Figure 3F**). A decision curve analysis plot based on cost-benefit and threshold probability further supported the stability and reliability of the model in practical application (**Figure 3G**). Collectively, ROC analysis demonstrated that the predictive model possessed excellent diagnostic efficacy in identifying BaP-associated EC, with an AUC of 0.985 (95% CI: 0.966-1.000, **Figure 3H**).

### 3.4 Validation of the prognostic model based on core genes

**Figure 4:**
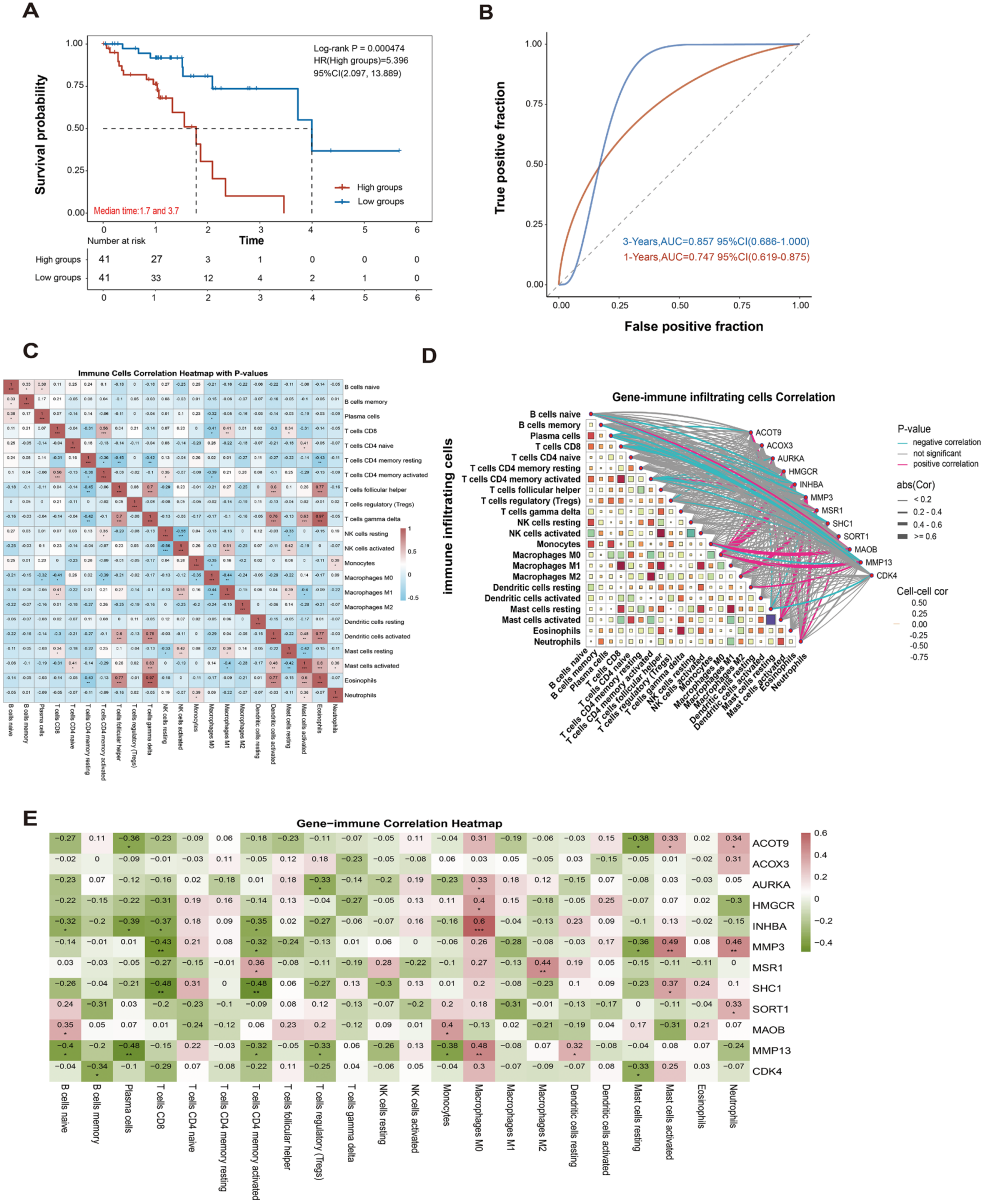
Survival and immune correlation analyses of the 12-gene model. **(A)** Kaplan-Meier analysis of overall survival was performed based on gene expression data from the TCGA-ESCC database. **(B)** ROC curves. **(C)** Heatmap of correlations among immune cell subsets. **(D, E)** Heatmaps illustrating the correlations between the 12 model genes and 22 immune cell subtypes.

Based on the 12-gene prognostic model constructed using the TCGA-ESCC cohort, Kaplan-Meier survival analysis revealed that patients in the high-risk group exhibited significantly poorer overall survival compared with those in the low-risk group (HR = 5.396, 95% CI: 2.097–13.889, log-rank P = 0.000474; **Figure 4A**). ROC curve analysis yielded AUC values of 0.747 (95% CI: 0.619-0.875) and 0.857 (95% CI: 0.686-1.000) for 1-year and 3-year survival, respectively (**Figure 4B**), indicating favorable discriminatory ability of the model.

### 3.5 Immune infiltration analysis of the core genes

The association characteristics between the 12 genes model and the tumor immune microenvironment were further explored. First, correlation analysis among immune cells revealed a complex interaction network of immune cells in the esophageal cancer tumor microenvironment (**Figure 4C**), with some cell subsets showing strong synergistic or antagonistic relationships. Specifically, gamma delta T cells were positively correlated with eosinophils and negatively correlated with activated memory CD4^+^ T cells. Gene−immune correlation analysis showed that the 12 genes model were significantly associated with the infiltration levels of various immune cells (**Figures 4D, E**). For example, genes including *SHC1* and *MMP3* were significantly negatively correlated with CD8^+^ T cell infiltration, while *INHBA* was strongly positively correlated with M0 macrophage infiltration, suggesting that these genes may participate in the formation of the immunosuppressive microenvironment by regulating effector T cell exhaustion and the enrichment of tumor−associated macrophages. In addition, multiple genes such as *MMP13* and *MAOB* also exhibited significant correlations with immune cell subsets including B cells and monocytes. Collectively, the 12−gene model in this study was closely related to the landscape of the esophageal cancer tumor immune microenvironment, mainly characterized by reduced infiltration of effector immune cells and enrichment of tumor−associated myeloid and stroma−related immune components.

### 3.6 SHAP analysis of key genes

**Figure 5:**
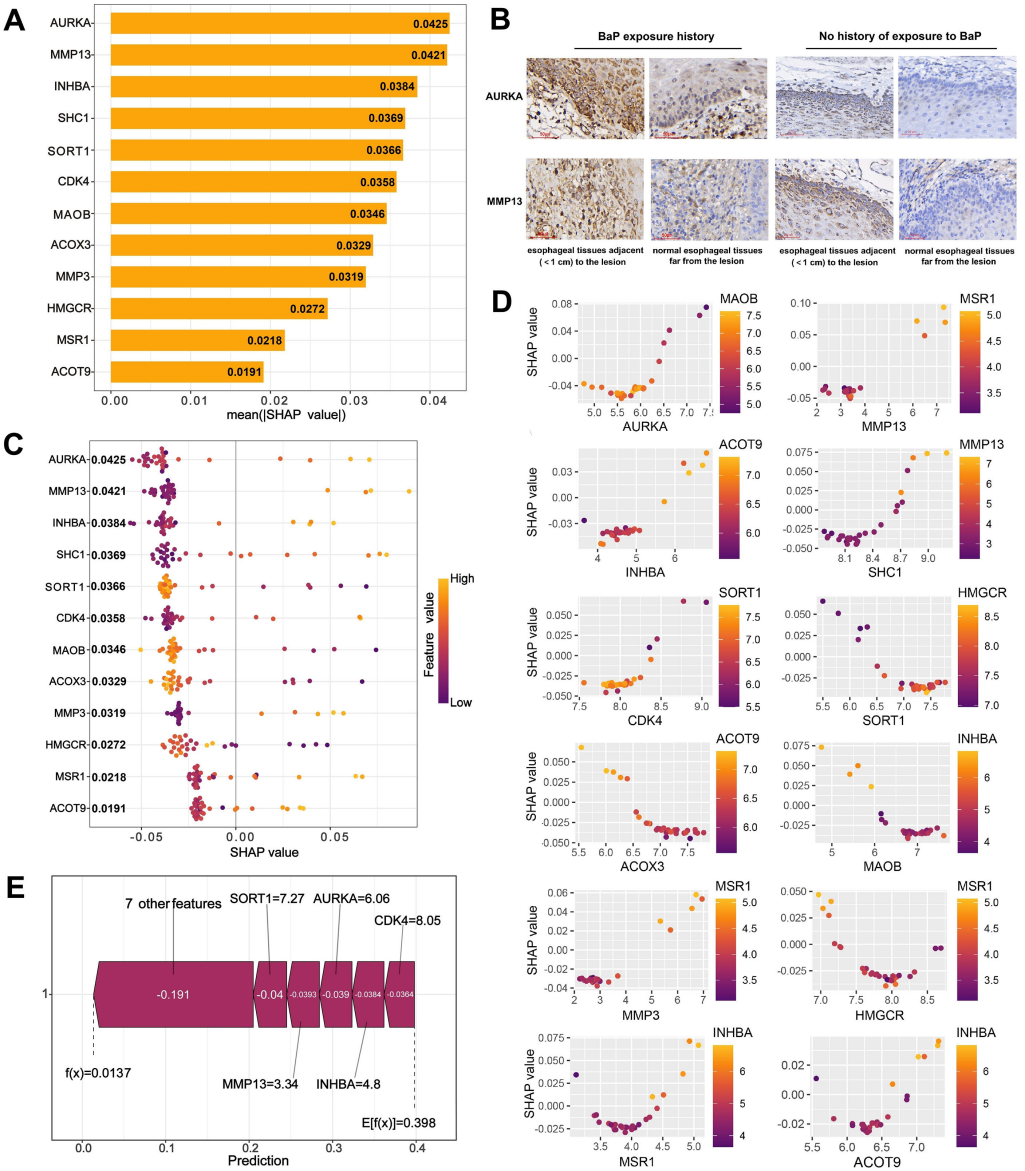
SHAP analysis reveals the contribution of model features. SHAP value quantifies each gene’s marginal influence on diagnostic prediction, positive values promote high-risk prediction while negative values reduce predicted risk. **(A)** Feature importance ranking bar chart sorted by absolute SHAP value. **(B)** Representative IHC staining of AURKA (top) and MMP13 (bottom) in esophageal tissues from BaP-exposed patients and non-exposed control subjects. **(C)** Violin plot displaying the distribution of gene expression under different conditions. **(D)** SHAP scatter plot presenting the SHAP values of key genes. **(E)** SHAP summary plot showing the overall direction and magnitude of contribution of each feature to the prediction results.

SHAP interpretability analysis further clarified the contribution of each feature gene to model prediction. Among them, *AURKA* (SHAP value=0.0425) and *MMP13* (0.0421) exerted the most significant effects on the prediction results and were identified as the key variables driving model discrimination (**Figure 5A**). In immunohistochemical analysis, high expression trends of *AURKA* and *MMP13* were observed in esophageal tissues adjacent to esophageal cancer lesions from patients with potential BaP exposure history, compared with normal esophageal tissues distant from the lesions (**Figure 5B**).

Furthermore, *HMGCR* and *ACOT9* exhibited expression level-dependent bidirectional regulatory patterns (**Figure 5C**). Notably, various potential nonlinear association patterns were revealed by the model: a synergistically enhancing positive correlation was observed between *MMP13* and *MSR1*; the protective effect of *ACOX3* was most prominent at high expression levels, whereas the strongest protective effects of *HMGCR* and *MSR1* were detected at moderate expression levels (**Figure 5D**).

Further force-directed analysis demonstrated that key genes including *AURKA*, *CDK4*, and *SORT1* mainly drove the model output through negative contributions, leading to a significant decrease in the predicted value from the baseline expectation E[f(x)]=0.398 to f(x)=0.0137 (**Figure 5E**).

### 3.7 Validation in single-cell transcriptomic datasets

**Figure 6:**
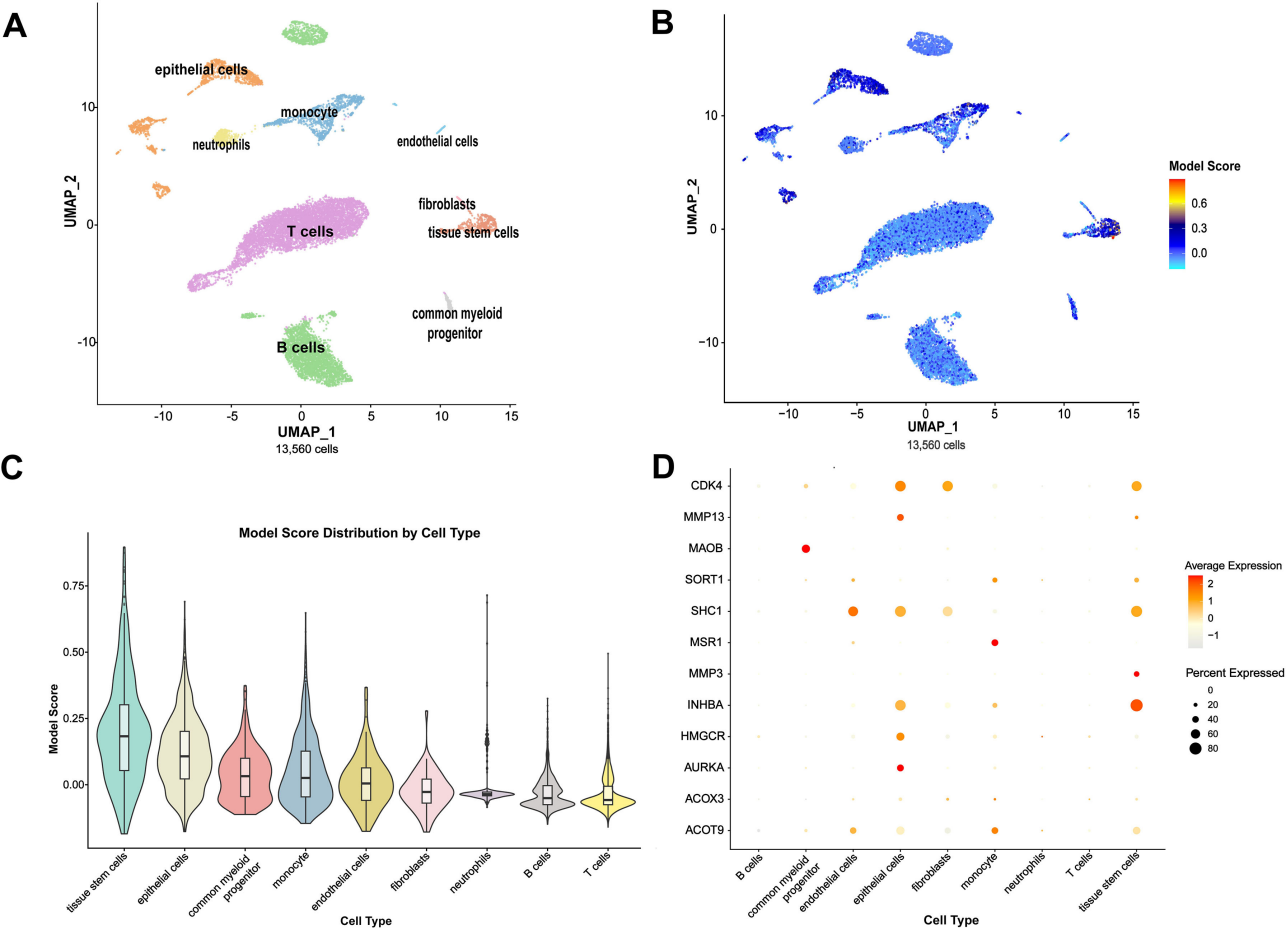
Single-cell transcriptome analysis. **(A, B)** UMAP visualization of RNA sequencing data from GSE196756 was performed, which was colored by cell type **(A)** and model score **(B)**, respectively. **(C)** Comparison of the distribution of model scores among different cell types was conducted. **(D)** Dot plot showing the expression levels and expression proportions of 12 core genes in different cell types.

Following the application of key genes to single-cell transcriptomic data, marked heterogeneity in model scores was observed among distinct cell populations. The highest median score was detected in tissue stem cells. Relatively high risk scores were also observed in myeloid cell populations, including common myeloid progenitors (CMP) and monocytes, indicating a potential correlation between high model risk score of myeloid progenitor cells and BaP exposure-related pro-tumorigenic characteristics. In contrast, low overall scores were exhibited by neutrophils and B cells, whereas moderate distributions were shown by fibroblasts and endothelial cells (**Figures 6A–C**).

At the single-gene expression level, distinct cell-type-specific expression patterns of key genes were observed (**Figure 6D**). The mitotic kinase *AURKA* was markedly upregulated in epithelial cell populations with high scores, supporting its key regulatory role in tumor cell proliferation and genome instability. *MAOB*, a pro-oxidative metabolic enzyme, was significantly upregulated in CMP cell populations with high scores, suggesting its potential regulatory role in the inflammatory microenvironment and abnormal differentiation by modulating monoamine substrate metabolism and reactive oxygen species (ROS) production. *INHBA*, a member of the TGF-β superfamily, was highly expressed in tissue stem cells, implying its potential function in stemness maintenance and tumor initiation. Furthermore, *MSR1* was prominently highly expressed in monocytes, further supporting its involvement in tumor-associated inflammation and the formation of the immunosuppressive micro environment.

### 3.8 Molecular docking validation of interactions between BaP and core genes

**Figure 7:**
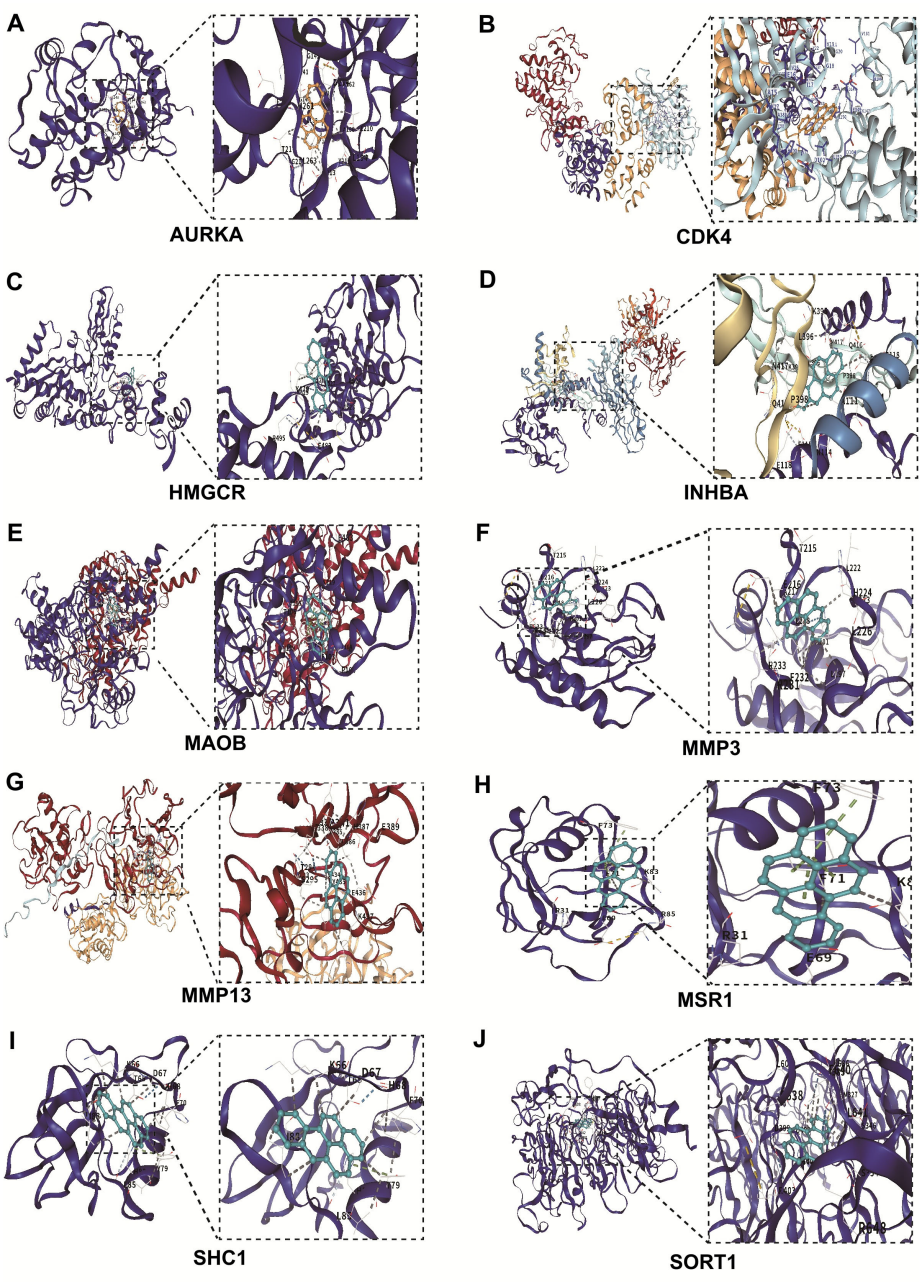
Molecular docking analysis of the interaction between BaP and core target proteins. **(A)** Docking results of AURKA with BaP. **(B)** Docking results of CDK4 with BaP. **(C)** Docking results of HMGCR with BaP. **(D)** Docking results of INHBA with BaP. **(E)** Docking results of MAOB with BaP. **(F)** Docking results of MMP3 with BaP. **(G)** Docking results of MMP13 with BaP. **(H)** Docking results of MSR1 with BaP. **(I)** Docking results of SHC1 with BaP. **(J)** Docking results of SORT1 with BaP.

To evaluate the potential binding ability between BaP and proteins encoded by the screened core target genes, comprehensive molecular docking analysis was performed. The results showed that strong binding affinities were observed between BaP and all 10 target proteins, with binding energies lower than -5 kcal/mol (**Table 1**), indicating that stable and spontaneous molecular interactions were formed. Visualization of the docked conformations (**Figures 7A–J**) further demonstrated that stable binding patterns were established in all BaP-protein complexes. Collectively, these results structurally supported that direct molecular interactions may exist between BaP and the EC-related core targets identified by machine learning.

**Table 1:** Binding energies of BaP with core target proteins.

Ligand	Receptor	Binding affinity (kcal/mol)	Interacting residues (chain labeled)
Benzo[a]pyrene	MAOB	-13.8	Chain B: GLY58 SER59 TYR60 PHE103 PRO104 ASN116 TRP119 LEU164 PHE168 LEU171 CYS172 TYR188 THR195 THR196 ILE198 ILE199 GLN206 LYS296 TYR326 LEU328 PHE343 TYR398 GLY434 TYR435 MET436 GLU483
Benzo[a]pyrene	AURKA	-11.2	Chain A: LEU139, GLY140, LYS141, GLY142, LYS143, PHE144, VAL147, ALA160, LYS162, LEU164, LEU169, VAL174, GLN177, LEU178, GLU181, LEU194, LEU210, GLU211, TYR212, ALA213, GLY216, THR217, LYS258, GLU260, ASN261, LEU263, ALA273, ASP274, GLY276, TRP277, THR288, LEU289, CYS290, GLY291
Benzo[a]pyrene	INHBA	-10.4	Chain A: ARG110 ALA111 ASN114 GLU115 GLU118 Chain B: THR394 LYS395 LEU396 PRO398 GLN416 ASN417 Chain D: THR394 LYS395 LEU396 PRO398 GLN416 ASN417 Chain C: ALA111 ASN114 GLU115 GLU118
Benzo[a]pyrene	MMP3	-10.1	Chain A: PRO160 GLY161 ASN162 VAL163 LEU164 ALA165 HIS166 GLU184 GLN185 THR191 GLY192 THR193 LEU197 VAL198 HIS201 GLU202 HIS205 HIS211 THR215 GLU216 ALA217 LEU218 TYR220 PRO221 LEU222 TYR223 HIS224 SER225 LEU226 ARG231 PHE232 ARG233
Benzo[a]pyrene	CDK4	-9.4	Chain C: ILE17 GLY18 VAL19 GLY20 ALA21 TYR22 VAL25 ALA38 LYS40 VAL77 PHE98 GLU99 HIS100 VAL101 ASP102 GLN103 ASP104 THR107 HIS143 ARG144 ASP145 GLU149 ASN150 LEU152 ALA162 ASP163 PHE164 LEU166 ALA167 TYR170 SER171 MET174 VAL179 VAL181 ARG186
Benzo[a]pyrene	MMP13	-8.8	Chain A: ILE293 THR294 SER295 GLY298 THR300 LEU311 PRO313 ALA337 TYR338 GLU339 PRO341 ALA385 VAL386 HIS387 PHE388 GLU389 VAL434 TYR435 GLU436 LYS437 ASN438 ILE468 TRP470
Benzo[a]pyrene	SORT1	-8.7	Chain A: GLN308 MET327 THR345 SER346 VAL352 TYR353 SER354 ASP399 GLN400 GLY401 GLY402 ARG403 CYS601 ILE602 LEU603 GLU638 ASP639 PHE640 LEU641 CYS642 ASP643 PHE644 GLY645 TYR646 TYR647 ARG648 GLN658 ASP665 ILE685 PRO686
Benzo[a]pyrene	SHC1	-7.5	Chain A: HIS53 LEU54 LEU55 LEU56 VAL57 ARG64 THR65 LYS66 ASP67 HIS68 PHE70 TYR79 HIS80 LEU85 PRO86 ILE87 ILE88 SER89
Benzo[a]pyrene	HMGCR	-7.4	Chain A: TYR479 GLU482 THR483 ARG495 MET523 GLY524 CYS527 GLU528 ASN529 VAL530
Benzo[a]pyrene	MSR1	-6.9	Chain A: ARG31 ARG35 LEU67 ASN68 GLU69 VAL70 PHE71 PHE73 LYS83 ARG85 GLN86 THR89 ARG90 ALA91

## 4 Discussion

In this study, multi-omics datasets were integrated to systematically dissect molecular signatures correlated with BaP exposure in esophageal cancer. Through target prediction screening and multi-algorithm machine learning, 12 core genes closely associated with BaP were identified, and their robust discriminatory capacity in diagnostic modeling was validated, supporting their potential utility as molecular biomarkers for risk stratification of BaP-relevant EC. Combined with single-cell transcriptomic profiling, cell-type-specific expression landscapes of these signature genes were characterized, with notable shifts in pathways governing cell cycle activity, metabolic homeostasis, and tumor microenvironment homeostasis captured across distinct epithelial subpopulations. Collectively, these multi-layered observations expand the systematic molecular landscape linked to environmental carcinogen-associated esophageal malignancy.

Functional enrichment analysis revealed that BaP-associated key genes were significantly enriched in biological processes including cell cycle regulation, hormone metabolism, protein kinase complex assembly, and serine hydrolase activity, which was highly consistent with previous findings on the carcinogenic and toxicological mechanisms of BaP (27–29). As a Group I carcinogen, BaP has been confirmed to be metabolically activated *in vivo* to form DNA adducts, and DNA damage and genomic instability are directly induced (19). Meanwhile, excessive ROS production is significantly promoted by BaP exposure, and sustained oxidative stress and inflammatory responses are triggered, which jointly contribute to tumorigenesis and progression (30–33). On this basis, the present study further demonstrated that enrichment results indicated that the 12 core genes converge on canonical oncogenic signaling axes such as cell cycle disorder, proteolysis, and metabolic reprogramming, but also highly enriched in core cancer pathways including MAPK/mTOR, p53, VEGF, and Toll-like receptor signaling pathways, highlighting coordinated pathway alterations correlated with BaP exposure in esophageal epithelial lesions. Cellular experiments in rat esophageal epithelial cells confirmed that the MAPK/ERK axis we identified mediates BaP-triggered oncogenic iNOS overexpression together with NF-κB signaling (34). Omics-based cellular assays in human esophageal cell lines have confirmed that short-term BaP exposure triggers metabolic and redox disorders to disrupt esophageal epithelial cell function (35), while functional *in vitro* studies of laryngeal and prostate tumors have demonstrated that BaP stimulation upregulates key oncogenes to drive malignant phenotypes (36, 37). Additionally, pan-cancer computational toxicology work identified shared pro-tumor signaling cascades induced by BaP across diverse epithelial-derived malignancies (38). Such cross-tumor observations increase the biological plausibility of our omics-derived correlation between BaP exposure and elevated *AURKA*/*MMP13* expression in esophageal lesions. Molecular docking simulations further identified favorable binding pockets between BaP and hub proteins *ACOX3*, *AURKA*, *CDK4* and *HMGCR*, confirming stable intermolecular interaction potentials at the structural level. Such ligand-protein binding interactions may interfere with physiological cycling of cell proliferation, lipid metabolism and proteolytic signaling, matching the aberrant proliferative and invasive phenotypes characteristic of esophageal carcinoma (39, 40).

In the present study, relatively high model risk scores were observed in tissue stem cells and myeloid cell populations, while lower scores were detected in neutrophils and B cells. Further gene expression localization analysis revealed that AURKA was specifically highly expressed in epithelial cells. Combined with its known roles in promoting cell proliferation and chromosomal instability across multiple malignancies, this gene may drive proliferative abnormalities within esophageal epithelial subpopulations (41–43). Published reports have linked AURKA expression to tumor immune cell infiltration profiles, suggesting a correlative relationship between this gene and immune microenvironment features (44). INHBA showed prominent expression in tissue stem cell subsets, consistent with previously reported links between INHBA and stemness-related pathways (45, 46). MSR1 was predominantly enriched in monocytes, matching prior work associating this gene with tumor-associated inflammatory signaling and immunosuppressive phenotypes (47, 48). Our single-cell transcriptomic observations revealed cell-type-specific expression patterns of these core genes, with distinct risk score distributions across stem, epithelial and myeloid compartments. Additional immune correlation analysis demonstrated that the 12 core genes correlate with the infiltration abundance of multiple immune cell subtypes in esophageal cancer, indicating potential links between these signature genes and an immunosuppressive tumor microenvironment. These transcriptome-level observations expand existing understanding of BaP-related carcinogenic pathways and highlight cell-type-specific expression features of candidate risk genes.

Furthermore, the diagnostic model constructed based on the 12 core genes exhibited excellent predictive performance (AUC = 0.985). Good clinical application potential was supported by nomogram, calibration curve, and decision curve analysis. SHAP analysis further revealed that *AURKA* and *MMP13* were key features with high contributions in the model, and expression level-dependent nonlinear effects were observed for multiple genes, reflecting their complex regulatory patterns during disease development. Collectively, these analyses position the 12-gene signature as a promising molecular panel for risk screening and early stratification of BaP-correlated esophageal cancer, while also serving as core molecular nodes tied to BaP toxic transcriptional responses.

Although the molecular characteristics of BaP-associated esophageal cancer were revealed from multiple dimensions in this study, several limitations still exist. Firstly, this study was mainly based on public databases and bioinformatic methods; all inferences about cell subpopulation crosstalk and microenvironmental regulation rely solely on omics correlation data, with underlying molecular mechanisms awaiting refined functional verification. Secondly, real environmental exposure involves mixed pollutants, while only BaP was analyzed here, leaving synergistic pollutant interactions uncharacterized. Thirdly, our retrospective IHC cohort adopted unified inclusion and exclusion criteria. Subjects with long-term exposure to cooking fumes, tobacco, grilled food or industrial dust were enrolled; those with other malignancies or benign esophageal lesions were excluded. Exposure types and approximate durations were recorded via questionnaires, while quantitative BaP metabolite detection was unavailable. Fourthly, although these core genes were filtered from BaP target pools, many of them are common oncogenic markers across cancers; parallel cell exposure tests using different carcinogens are required to further confirm their specificity to BaP-associated esophageal carcinogenesis. Finally, all BaP target candidates were predicted via three in silico databases with inherent false-positive risks, and multi-layer filtering and molecular docking could not substitute cellular and biochemical validation of authentic ligand-protein interactions.

## 5 Conclusion

Multi-omics integration and machine learning screening identified 12 core genes with strong molecular correlation to BaP-associated esophageal malignancy. Structural molecular docking validated stable intermolecular binding between BaP and key proteins including *AURKA*, *MMP13* and *INHBA*, with these signature genes linked to dysregulated cell cycle signaling, metabolic homeostasis shifts and stem cell transcriptional programs in esophageal lesions. The constructed 12-gene risk prediction model exhibits favorable predictive efficiency, which can provide a basis for the early warning and risk assessment of environmentally related esophageal cancer, and also offer evidence for targeted intervention in BaP-associated carcinogenic processes.

## Data Availability

The original contributions presented in the study are included in the article/supplementary material. Further inquiries can be directed to the corresponding author.
